# Congenital Pouch Colon in a Neonate

**DOI:** 10.12669/pjms.38.ICON-2022.5771

**Published:** 2022-01

**Authors:** Sana Niaz, Sahira Naz, Rumaissa Abdul Raziq

**Affiliations:** 1 Sana Niaz, MBBS, FCPS, Department of Neonatology, Indus Hospital & Health Network, Karachi, Pakistan; 2 Sahira Naz, MBBS, FCPS, Department of Pediatrics, Indus Hospital & Health Network, Karachi, Pakistan; 3 Rumaissa Abdul Raziq, BDS, Indus Hospital Research Center, Indus Hospital & Health Network, Karachi, Pakistan

**Keywords:** Congenital pouch colon, Anorectal malformation, Pouch colon syndrome, Invertogram

## Abstract

Congenital Pouch Colon (CPC) is a rare anorectal malformation (ARM) in which a part of or the entire colon is replaced by pouch-like dilatation. Males are more likely to be diagnosed with the condition compared to females. The highest incidence of the disease is in South Asia, with a significant number of cases reported from India. Early diagnosis can be made when there are hypoechogenic lesions on antenatal ultrasound scans. We report a case of a neonate with routine antenatal scans who presented with a distended abdomen and inability to pass feces. The diagnosis was made in the early neonatal period, followed by surgical management.

## INTRODUCTION

Congenital Pouch Colon (CPC), also known as short colon syndrome, is a rare anorectal malformation predominantly seen in people from Northern India, with a reported incidence of 5-10%.^1^ The condition occurs predominantly in males with a ratio varying from 2.25:1 to 7:1.^2^,^3^ B Mirza conducted a retrospective study with a sample of 21 children. The study aimed to decipher the incidence of CPC anorectal malformation in Pakistan. Out of 21, 18 were suffering from CPC with high ARM and three with low ARM. The study reported an incidence of 6.73%.^4^

In CPC, part or whole of the colon is replaced by a dilated pouch which communicates via a fistula with the genitourinary tract.^1^ It is classified into five types depending on the length of colonic involvement (Table-I). Associated genitourinary anomalies may accompany and include hydronephrosis, hydroureteronephrosis, renal agenesis, vesicoureteral reflux, cryptorchidism, and hypospadias. Other conditions may also co-exist like congenital heart disease, sacral agenesis, Meckel’s diverticulum, and colon duplication.^4^ Treatment involves surgical management which in turn depends on the length of colonic involvement and type of CPC. Here we report a case of a 2-day-old male child with type-1 congenital colonic pouch.

**Table I:** Types of Congenital Pouch Colon (CPC).

Types of CPCS	Classification
Type-1	Normal colon absent and ileum opens into pouch colon
Type-2	Ileum opens into a normal cecum which opens into the pouch colon
Type-3	Normal ascending colon and transverse colon opens into the pouch colon
Type-4	Normal colon with recto-sigmoid pouch
Type-5	Double pouch colon with short normal inter-positioned colon segment

## CASE REPORT

A 2-day-old male infant was brought to the Emergency Department of the Indus Hospital and Health Network, with the complaint of failure to pass stool since birth. Abdominal distension was observed on the 1^st^ day of life by parents. They also noted meconium in urine. Medical history revealed that he was delivered at full term by normal vaginal delivery with no antenatal and postnatal complications; to a third gravida mother with two alive and healthy children. The mother’s antenatal scans were normal. Furthermore, there was no history of premature rupture of membrane or gestational diabetes mellitus. Family history revealed a non-consanguineous marriage between parents with no history of gastrointestinal anomalies, sudden infant death or miscarriages.

**Fig.1:**
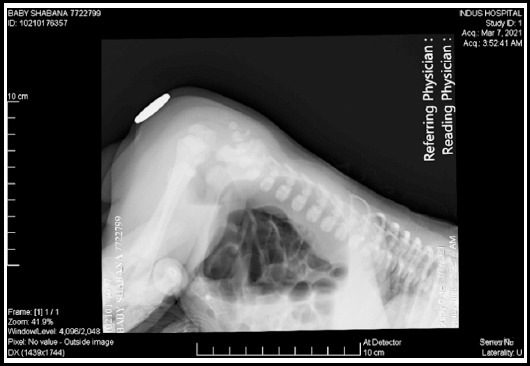
Invertogram.

**Fig.2:**
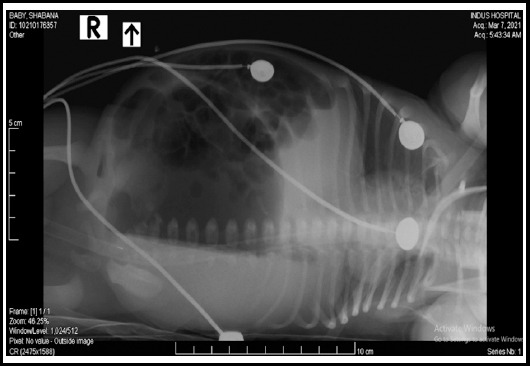
Radiograph abdomen (lateral decubitus view).

On physical examination, the baby’s weight was 2.9 kg; he appeared dehydrated, lethargic, with gross abdominal distension and had no anal opening. A nasogastric tube was passed to rule out associated tracheoesophageal fistula, which passed effortlessly and was visualized in the stomach on a radiograph. Moreover, an invertogram (Fig.1) and abdominal radiograph (Fig.2) were performed, which confirmed a high type of anorectal malformation and an air-fluid level with a large bowel loop, respectively. Ultrasound abdomen and echocardiography were done to rule out other associated anomalies, but were normal.

After initial stabilization, appropriate antibiotic initiation, and anesthesia fitness evaluation, he was shifted to the operation theater for a laparotomy. Perioperative findings were suggestive of a type 1 pouch colon and no normal colon could be identified. A large thick muscular fistula communicating with the bladder (posterior wall) was identified and an ileostomy was performed 25cm above the rectum. Diagnosis of “congenital pouch colon associated with imperforate anus and recto-vesical fistula” was made. Postoperatively, the baby remained vitally stable; stoma started to function, feeding was started and progressively increased, and later direct breastfeeding was started which he tolerated well. Following a detailed assessment, the attending physician and surgeon counseled the infant’s parents regarding his diagnosis, likely prognosis, follow-up and home care plans and future surgical plan. The baby was discharged home on out-patient follow up.

## DISCUSSION

Early diagnosis of the syndrome plays a significant role in reducing mortality and morbidity. In our case, the baby was diagnosed with CPC, a rare condition in the early neonatal period. Due to low incidence and therefore awareness in Pakistan and no family history, it may have been missed resulting in a poor outcome.

In 1912, Spriggs originally described Congenital Pouch Colon in a London Hospital Museum, on a specimen that manifested absence of half of the colon and rectum.^5^ The name, Pouch colon syndrome, was suggested by Narsimha Rao et al. in 1984.^6^ Since then, different theories have been conducted to unravel its etiology. Wakhlu et al postulated that it is due to the combination of defective development and failure of rotation of the gut during intrauterine life.^7^ Some have proposed vascular impairment as the cause. Genetic factors like Wnt, NOTCH and Hedgehog gene mutation and environmental factors like vitamin B and iodine deficiency are also associated with CPC.^8^ The most straightforward classification of CPC was proposed by Saxena and Mathur (Table-I) in 2008. It classified CPC into five types depending on the length of colonic involvement. Another classification by Gupta DK classified CPC as complete and incomplete depending upon the length of the colon left to perform the pull-through procedure.^1^

In contrast with a normal colon, CPC varies anatomically, histologically, and functionally. On gross examination, the normal anatomical findings of *Taenia coli*, smooth muscle, haustration, and appendices epiploicae were absent along with a small pouch with rudimentary mesentery, indicative of the true CPC. The vascular supply of the pouch is from the branches of the superior mesenteric artery. Histopathologically, the muscle wall of the pouch is un-differentiated between the inner circular muscles and outer longitudinal muscles. Mature ganglion cells present in most cases; however, giant ganglia are also present in 10% of cases.^1^

The clinical presentation of CPC can be made early in the neonatal period. However, it may vary due to associated factors like the gender of the baby, type of CPC, and size of the fistulous connection between the colon and genitourinary tract. In most of the cases, males may present with imperforate anus and abdominal distension, and in 50 % cases, fecaluria may be seen. On the other hand, females often present with abdominal distension, but sometimes urinary incontinence, constipation, and enterocolitis may follow.^1^

For better diagnosis, preoperative radiographic investigations, like an erect skiagram and an invertogram are required which generally show a large air-fluid level in the bowel loop; a suggestive radiographic finding of meconium.^4^ For co-existing anomalies ultrasound of abdomen and echocardiograph along with voiding cystourethrography must be performed. Out of the many complications found to be associated with CPC post-operatively, diarrhea, urinary tract infections, failure to gain weight, bowel stenosis, rectal or bowel prolapse, wound dehiscence, enterocolitis, and septicemia are among the most common.^1^ The prognosis of the syndrome depends on several factors, like type of CPC, age at presentation, presence of associated anomalies, and presence of septicemia and gut perforation.

**Fig.3:**
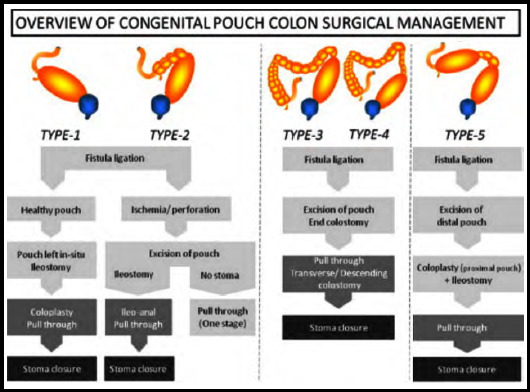
Surgical Management of CPC.

The standard treatment for patients with CPC is resection of the pouch with abdominoperineal pull-through in multiple stages.^9^ The surgical management of CPC is explained in Fig.3.^10^

The baby in our case had presented with typical CPC features of abdominal distention and inability to pass feces since birth. A pre-operative diagnostic invertogram was performed along with an abdominal ultrasound to arrive at the correct diagnosis. Confirmatory diagnosis of type 1 CPC was made based on per-operative findings. The parents were counseled regarding the child’s condition to ensure timely follow-up and further staged surgical plans.

## CONCLUSION

As CPC is a rare syndrome, it can be missed by obstetricians in the prenatal period. Detailed antenatal and postnatal evaluation leads to prompt diagnosis and consequently minimal complications. It also allows early screening and management of associated conditions. Timely diagnosis and management have a significant impact on overall prognosis and reduction of associated mortality.

### Authors Contribution:

**SNI:** conception of idea, designing the study, literature search, data compilation, manuscript writing and review.

**SNA:** conception of idea, literature search, manuscript writing, review and is responsible for integrity of the study.

**RAR:** Literature search, manuscript writing and review.
